# Supplementary material for: Integrating Genomic and Chromosomal Data: A Cytogenetic Study of Transancistrus santarosensis (Loricariidae: Hypostominae) with Characterization of a ZZ/ZW Sex Chromosome System
Source: Genes (Basel). 2023 Aug 22;14(9):1662. doi: 10.3390/genes14091662 (PMC10531053; doi:10.3390/genes14091662)
Supplement: Supplementary file 1 [file genes-14-01662-s001.zip › TableS1-final.pdf]

Table S1. *Transancistrus santarosensis*. Summary of the information on collection date and site, sex (M = male, F = Female, I = Indeterminate) and analysis of the specimens. *Chaetostoma bifurcum* individuals used in molecular analysis are also included. COI haplotype is indicated. Voucher numbers refer according to the Universidad Technica de Machala catalogue (UTMACH) or the Instituto Nacional de Biodiversidad collection (MECN-DP)

**Individual**	Genus/species	**Collection site**	**Date of collection**	**Sex**	**Cytogenet**	**COI Hp**	**Voucher n.**
	Transancistrus						
155	*T. santarosensis*	Palenque	24/02/2016	M	X	Ts1	UTMACH-155
156	*T. santarosensis*	Palenque	24/02/2016	I	X	Ts1	UTMACH-156
157	*T. santarosensis*	Palenque	24/02/2016	M	X	Ts1	UTMACH-157
158	*T. santarosensis*	Río Dos Bocas	24/02/2016	M	-	Ts2	UTMACH-158
443	*T. santarosensis*	Río Dos Bocas	1/07/2022	I	X	-	UTMACH-443
444	*T. santarosensis*	Río Dos Bocas	1/07/2022	I	X	-	MECN-DP 4960
445	*T. santarosensis*	Río Dos Bocas	1/07/2022	I	X	-	MECN-DP 4960
446	*T. santarosensis*	Río Dos Bocas	1/07/2022	I	X	-	MECN-DP 4960
447	*T. santarosensis*	Río Dos Bocas	1/07/2022	M	X	-	MECN-DP 4960
448	*T. santarosensis*	Río Dos Bocas	1/07/2022	F	X	-	MECN-DP 4960
452	*T. santarosensis*	Río Dos Bocas	2/09/2022	I	X	Ts2	UTMACH-452
453	*T. santarosensis*	Río Dos Bocas	2/09/2022	I	X	-	UTMACH-453
457	*T. santarosensis*	Río Dos Bocas	5/09/2022	F	X	Ts2	UTMACH-457
458	*T. santarosensis*	Río Dos Bocas	7/09/2022	F	X	Ts2	UTMACH-458
460	*T. santarosensis*	Palenque	11/09/2022	F	X	-	UTMACH-460
464	*T. santarosensis*	Palenque	11/09/2022	F	X	-	UTMACH-464
465	*T. santarosensis*	Palenque	11/09/2022	F	X	Ts1	UTMACH-465
466	*T. santarosensis*	Palenque	11/09/2022	F	X	Ts1	UTMACH-466
467	*T. santarosensis*	Palenque	11/09/2022	I	X	Ts1	UTMACH-467
468	*T. santarosensis*	Rio Dos Bocas	11/09/2022	I	X	-	UTMACH-468
469	*T. santarosensis*	Rio Dos Bocas	11/09/2022	F	X	-	UTMACH-469
470	*T. santarosensis*	Rio Dos Bocas	06/01/2023	I	X	-	UTMACH-470
471	*T. santarosensis*	Rio Dos Bocas	06//01/2023	F	X	-	UTMACH-471
472	*T. santarosensis*	Rio Dos Bocas	09/01/2023	I	X	-	UTMACH-472
474	*T. santarosensis*	Rio Dos Bocas	12/01/2023	I	X	-	UTMACH-474
	Chaetostoma						
437	*C. bifurcum*	Rio Dos Bocas	30/06/2022	I	-	Cb1	UTMACH-437
439	*C. bifurcum*	Rio Dos Bocas	30/06/2022	F	-	Cb1	UTMACH-439
461	*C. bifurcum*	Cascada Río Blanco	20/12/2022	M	-	Cb1	UTMACH-461
462	*C. bifurcum*	Cascada Río Blanco	20/12/2022	I	-	Cb1	UTMACH-462
