# Supplementary material for: Integrating Genomic and Chromosomal Data: A Cytogenetic Study of Transancistrus santarosensis (Loricariidae: Hypostominae) with Characterization of a ZZ/ZW Sex Chromosome System
Source: Genes (Basel). 2023 Aug 22;14(9):1662. doi: 10.3390/genes14091662 (PMC10531053; doi:10.3390/genes14091662)
Supplement: Supplementary file 1 [file genes-14-01662-s001.zip › TableS2.pdf]

Table S2. GenBank Accession Number and sampling area of COI sequences used in phylogenetic analyses

Species	A.N.	Sampling area
*Chaetostoma sp1_*	EU359409	Unknown
*Chaetostoma sp2_*	EU359410	Unknown
Chaetostoma fischeri	MG936828	Panama: Rio Chagres
Chaetostoma fischeri	MG936829	Panama: Rio Acla
Chaetostoma fischeri	MG936830	Panama: Rio Tuira, Rio Yape
Chaetostoma fischeri	MG936831	Panama: Rio Tuira, Rio Membrillo
Chaetostoma fischeri	MG936832	Panama: Rio Bayano, Rio Aguas Claras
Chaetostoma fischeri	MG936833	Panama: Rio Bayano, Rio Ipeti
Leptoancistrus canensis	MG937046	Panama: Rio Chagres
Leptoancistrus canensis	MG937047	Panama: Rio Indio
Leptoancistrus canensis	MG937048	Panama: Rio Cocle del Sur
Leptoancistrus canensis	MG937049	Panama: Rio Bayano, Rio Aguas Claras
Ancistrus clementinae	OQ132522	Ecuador: Rio Palenque, Rio La Moquillada
Ancistrus clementinae	OQ132523	Ecuador: Rio Palenque
