# Supplementary figures and images for: Integrating Genomic and Chromosomal Data: A Cytogenetic Study of Transancistrus santarosensis (Loricariidae: Hypostominae) with Characterization of a ZZ/ZW Sex Chromosome System
Source: Genes (Basel). 2023 Aug 22;14(9):1662. doi: 10.3390/genes14091662 (PMC10531053; doi:10.3390/genes14091662)

**Figure S1.** NJ (a) and ML (b) phylogenetic trees. Node support values higher than 75% are shown

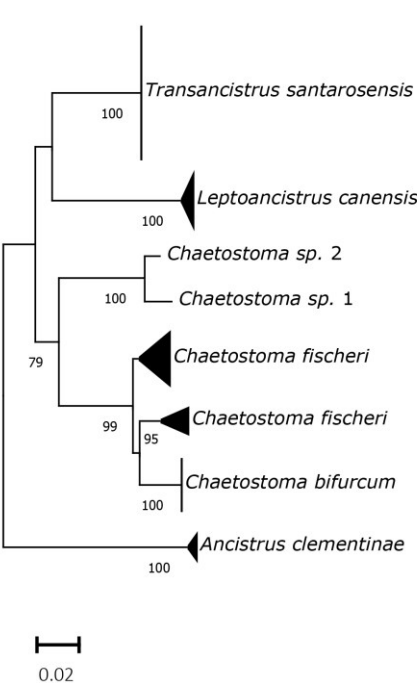

a)

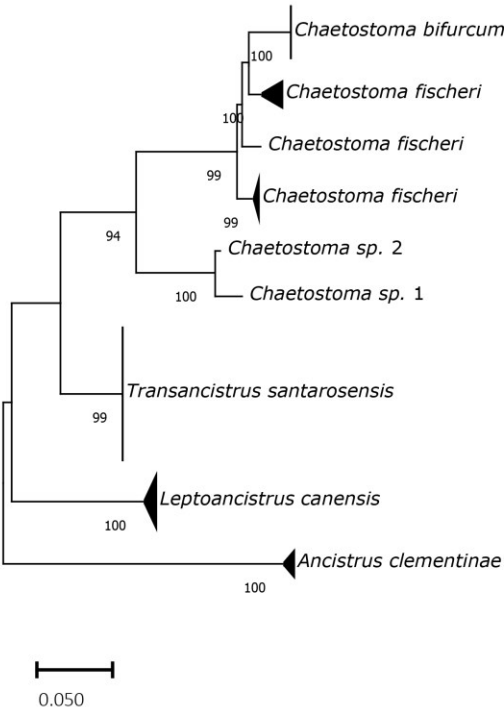

b)

Supplement: Supplementary file 1 [file genes-14-01662-s001.zip › FigS1.pdf]
